# Supplementary material for: A systematic review and meta-analysis of randomized controlled trials for physical activity among colorectal cancer survivors: directions for future research
Source: PeerJ. 2025 Jan 31;13:e18892. doi: 10.7717/peerj.18892 (PMC11789654; doi:10.7717/peerj.18892)
Supplement: Supplemental Information 1 [file peerj-13-18892-s001.docx]

**Title：**A systematic review and meta-analysis of randomized controlled trial for physical activity among the colorectal cancer survivors: directions for future research

**Data of total physical activity levels among the included studies (n=9)**

| author | year | n.e | mean.e | sd.e | n.c | mean.c | sd.c |
| --- | --- | --- | --- | --- | --- | --- | --- |
| Falz | 2023 | 62 | 124 | 141 | 60 | 114 | 111 |
| Leach | 2023 | 13 | 2679.5 | 330.6 | 12 | 3127.1 | 1768.4 |
| Cadmus-Bertram | 2019 | 24 | 1463 | 489 | 23 | 1343 | 395 |
| Kim | 2019 | 37 | 332.6 | 306.1 | 34 | 133.8 | 227.8 |
| Lee | 2018 | 38 | 571.1 | 382.4 | 34 | 309.4 | 196.7 |
| Van Waart(1) | 2018 | 7 | 97.7 | 38 | 8 | 120.6 | 46 |
| Van Waart(2) | 2018 | 6 | 113.4 | 48.1 | 8 | 120.6 | 46 |
| Courneya | 2016 | 106 | 1926 | 1842 | 105 | 1302 | 1212 |
| Backman | 2014 | 4 | 14 | 1.4 | 4 | 12.5 | 2.4 |
| Ligibel | 2012 | 48 | 54.5 | 142 | 51 | 14.6 | 117.2 |

**Note:** n.e = sample size of experimental group; mean.e = mean of total physical activity levels in experimental group; sd.e = standard deviation of experimental group; n.c = sample size of control group; mean.c = mean of total physical activity levels in control group; sd.c = standard deviation of control group.

**Code of meta-analysis conducted in R and RStudio**

#1 install.packages("forestplot")

#2 install.packages("ggplot2")

#3 install.packages("meta")

#4 install.packages("theme")

#5 library(forestplot)

#6 library(ggplot2)

#7 library(meta)

#8 library(theme)

#9 data.<-read.csv("C:/Users/25471/Desktop/PA.csv")

#10 data.

#11 m1<-metacont(text.study="Study or Subgroup",n.e,mean.e,sd.e,n.c,mean.c,sd.c,sm="SMD",overall=TRUE,

data=data.,studlab = paste(data.$author,data.$year,sep="-"))# *Meta-analysis*

#12 m1

#13 settings.meta("Revman")

#14 forest(m1,print.subgroup.labels =TRUE,pooled.totals = TRUE,overall = TRUE,label.left = "Favours[control]", label.right = "Favours[experimental]",leftlabs = c("Study or Subgroup", "Total","Mean","SD","Total","Mean","SD"), family="sans",fontsize =9.5,lebel.b="Intervention group",label.c="Control",Iwd=2,col.diamond.common ="maroon", col.diamond.lines.common= "maroon",col.diamond.random ="lightslategray", col.diamond.lines.random = "lightslategray",col.square = "skyblue", col.study = "lightslategray",col.subgroup="black",lty.common= 4,plotwidth = "8cm", colgap.forest.left = "1cm",colgap.forest.right = "1cm",just.forest="right", colgap.left = "0.5cm",colgap.right = "0.5cm",digits.sd = 2)+ theme(text = element_text(family = "Times New Roman"))

#15 funnel(m1)

#16 metainf(m1,pooled = "common")

#17 metabias(m1,k.min=2,method.bias = "linreg")

#18 m1<-metacont(text.study="Study or Subgroup",n.e,mean.e,sd.e,n.c,mean.c,sd.c,sm="SMD",byvar=subgroup,overall=TRUE,

data=data.,studlab = paste(data.$author,data.$year,sep="-"))# *Subgroup-analysis*

#19 forest(m1,print.subgroup.labels =TRUE,pooled.totals = TRUE,overall = TRUE,label.left = "Favours[control]", label.right = "Favours[experimental]",leftlabs = c("Study or Subgroup", "Total","Mean","SD","Total","Mean","SD"), family="sans",fontsize =9.5,lebel.b="Intervention group",label.c="Control",Iwd=2,col.diamond.common ="maroon", col.diamond.lines.common= "maroon",col.diamond.random ="lightslategray", col.diamond.lines.random = "lightslategray",col.square = "skyblue", col.study = "lightslategray",col.subgroup="black",lty.common= 4,plotwidth = "8cm", colgap.forest.left = "1cm",colgap.forest.right = "1cm",just.forest="right", colgap.left = "0.5cm",colgap.right = "0.5cm",digits.sd = 2)+ theme(text = element_text(family = "Times New Roman"))
