# Supplementary material for: A systematic review and meta-analysis of randomized controlled trials for physical activity among colorectal cancer survivors: directions for future research
Source: PeerJ. 2025 Jan 31;13:e18892. doi: 10.7717/peerj.18892 (PMC11789654; doi:10.7717/peerj.18892)
Supplement: Supplemental Information 5 [file peerj-13-18892-s005.docx]

Table S1 Search strategies

| **Date of search** | **Databases** | **Search strategy** | **Search results** |
| --- | --- | --- | --- |
| 3/1/2024 | Cochrane Library | ("physical activity intervention"[Title/Abstract/Keyword] OR "physical activity"[Title/Abstract/Keyword] OR "exercise"[Title/Abstract/Keyword] OR "aerobic exercise"[Title/Abstract/Keyword] OR "resistance exercise"[Title/Abstract/Keyword]) AND ("colorectal cancer survivors"[Title/Abstract/Keyword] OR "colorectal cancer patients"[Title/Abstract/Keyword] OR "colon cancer"[Title/Abstract/Keyword] OR "rectal cancer"[Title/Abstract/Keyword]) AND ("randomized controlled trial"[Title/Abstract/Keyword] OR "randomized"[Title/Abstract/Keyword] OR "RCT"[Title/Abstract/Keyword]) AND [2010-2024]/py | 653 |
| 3/1/2024 | Embase | ('physical activity intervention' OR (physical AND ('activity'/exp OR activity) AND ('intervention'/exp OR intervention)) OR 'physical activity'/exp OR 'physical activity' OR (physical AND ('activity'/exp OR activity)) OR 'exercise'/exp OR exercise OR 'aerobic exercise'/exp OR 'aerobic exercise' OR (aerobic AND ('exercise'/exp OR exercise)) OR 'resistance exercise'/exp OR 'resistance exercise' OR (('resistance'/exp OR resistance) AND ('exercise'/exp OR exercise))) AND ('colorectal cancer'/exp OR 'colorectal cancer' OR (colorectal AND ('cancer'/exp OR cancer)) OR 'colorectal neoplasm'/exp OR 'colorectal neoplasm' OR (colorectal AND ('neoplasm'/exp OR neoplasm)) OR 'colon cancer'/exp OR 'colon cancer' OR (('colon'/exp OR colon) AND ('cancer'/exp OR cancer)) OR 'rectal cancer'/exp OR 'rectal cancer' OR (rectal AND ('cancer'/exp OR cancer))) AND ('randomized controlled trial'/exp OR 'randomized controlled trial' OR (randomized AND controlled AND ('trial'/exp OR trial)) OR randomized OR rct) AND [randomized controlled trial]/lim AND [2010-2024]/py | 186 |
| 3/1/2024 | PubMed | ("physical activity intervention"[Title/Abstract] OR "physical activity"[Title/Abstract] OR "exercise"[Title/Abstract] OR "aerobic exercise"[Title/Abstract] OR "resistance exercise"[Title/Abstract]) AND (("colorectal cancer survivors"[Title/Abstract] OR "colorectal cancer patients"[Title/Abstract] OR "colon cancer"[Title/Abstract] OR "rectal cancer"[Title/Abstract]) AND ("randomized controlled trial"[Title/Abstract] OR "randomized"[Title/Abstract] OR "RCT"[Title/Abstract])) AND ((randomized controlled trial[Filter]) AND (humans[Filter]) AND (2010/1/1:2024/3/1[pdat]) AND (chinese[Filter] OR english[Filter])) | 224 |
| 3/1/2024 | Scopus | TITLE-ABS-KEY ((physical AND activity AND intervention OR physical AND activity OR exercise OR aerobic AND exercise OR resistance AND exercise) AND (colorectal AND cancer OR colorectal AND neoplasm OR colon AND cancer OR rectal AND cancer) AND (randomized AND controlled AND trial OR randomized OR rct)) AND PUBYEAR > 2009 AND PUBYEAR < 2025 AND (LIMIT-TO (LANGUAGE, "English")) | 163 |
| 3/1/2024 | Web of Science | (("physical activity intervention"[Title/Abstract] OR "physical activity"[Title/Abstract] OR "exercise"[Title/Abstract] OR "aerobic exercise"[Title/Abstract] OR "resistance exercise"[Title/Abstract]) AND ("colorectal cancer survivors"[Title/Abstract] OR "colorectal cancer patients"[Title/Abstract] OR "colon cancer"[Title/Abstract] OR "rectal cancer"[Title/Abstract]) AND ("randomized controlled trial"[Title/Abstract] OR "randomized"[Title/Abstract] OR "RCT"[Title/Abstract])) AND ((randomized controlled trial[Filter]) AND (2010/1/1:2024/3/1[pdat]) AND (english[Filter]) ) | 626 |
| 3/1/2024 | China National Knowledge Infrastructure | ("physical activity intervention"[Subject] OR "physical activity"[Subject] OR "sports"[Subject] OR "exercise"[Subject]) AND ("colorectal cancer survivors"[Subject] OR "colorectal cancer patients"[Subject] OR "colon cancer"[Subject] OR "rectal cancer"[Subject]) AND ("randomized controlled trial"[Subject]) AND ((randomized controlled trial [Filter]) AND (2010/1/1:2024/3/1[pdat]) | 86 |
| 3/1/2024 | Wan Fang Data | (physical activity OR sports OR exercise OR aerobic exercise OR resistance exercise) AND (colorectal cancer survivors OR colorectal cancer patients OR colon cancer OR rectal cancer) AND (randomized controlled trial OR randomized OR RCT) AND Publish time: [2010/1/1: 2024/3/1] | 48 |
