# Supplementary figures and images for: A systematic review and meta-analysis of randomized controlled trials for physical activity among colorectal cancer survivors: directions for future research
Source: PeerJ. 2025 Jan 31;13:e18892. doi: 10.7717/peerj.18892 (PMC11789654; doi:10.7717/peerj.18892)

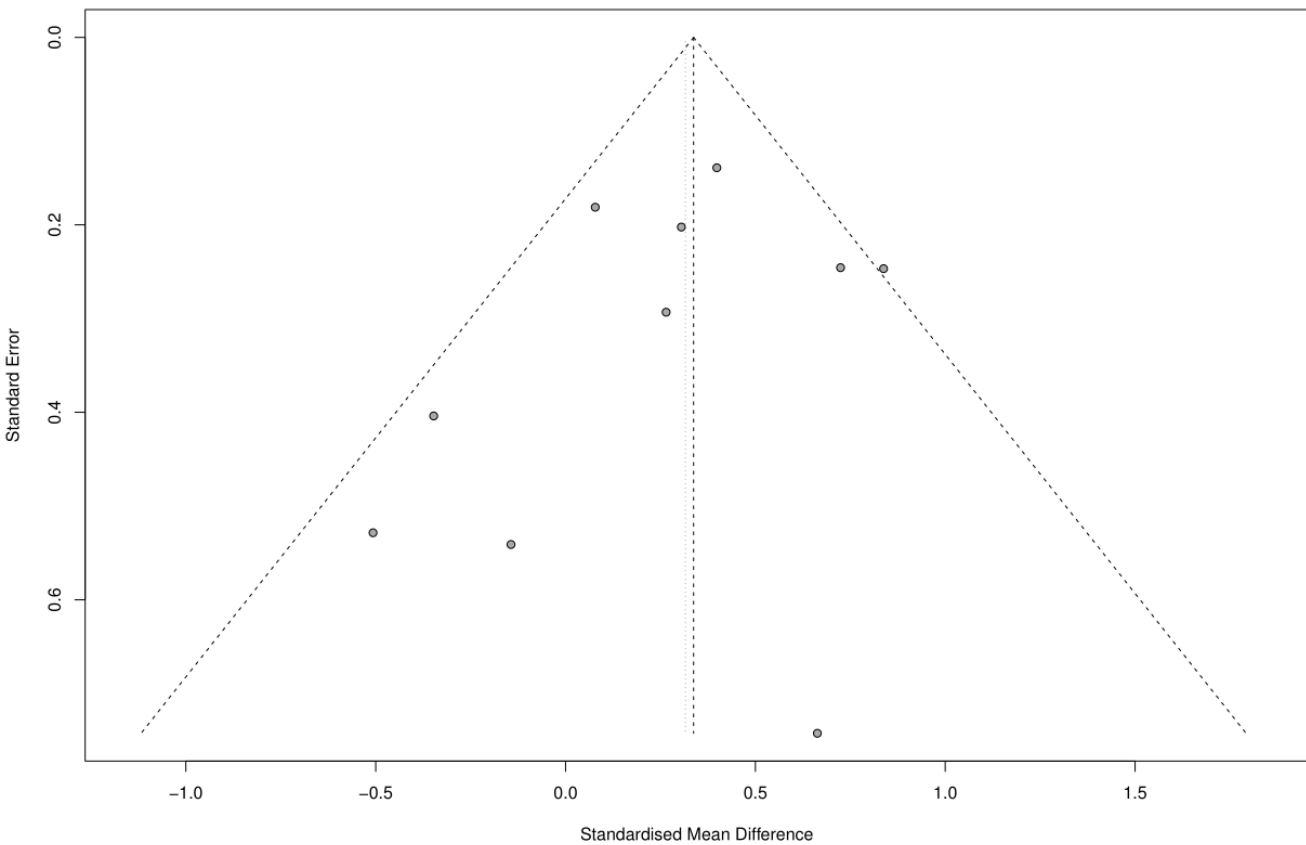

Supplement: Supplemental Information 6 — Note: There was no significant publication bias among the included studies. [file peerj-13-18892-s006.pdf]
